# As the Growing Season Progresses, the Key Driving Factor of Vegetation Growth Shifts From Spring Phenology to Temperature in the Cross‐Border‐Region of Northeast Asia

**DOI:** 10.1002/ece3.71384

**Published:** 2025-05-08

**Authors:** Lujie Zhao, Jihao Zhang, Xiao Huang, Duqi Liu, Zhen Xu, Guishan Cui

**Affiliations:** ^1^ College of Integration Science Yanbian University Yanji China; ^2^ College of Geography and Ocean Sciences Yanbian University Yanji China

**Keywords:** climate change, CRCDR, Northeast Asia, vegetation growth, vegetation phenology

## Abstract

Spring vegetation phenology reflects the dynamics of ecosystems and the status of vegetation growth. Earlier spring phenology can promote vegetation growth by extending the length of the vegetation growing season, thus improving the productivity and carbon sink function of terrestrial ecosystems. However, the stage‐specific effects of spring phenology and climate change on vegetation growth are yet to be effectively explained. Taking the Cross‐border Region of China, Democratic People's Republic of Korea, and Russia (CRCDR) as an example, we utilized the Normalized Difference Vegetation Index (NDVI) as a proxy for vegetation growth and extracted the start date of the growing season (SOS) from NDVI to characterize spring phenology and explored the relative importance of SOS and climate factors on vegetation growth. Results indicate that from 2001 to 2020, the SOS in the CRCDR region advanced at a rate of 0.22 days per year, and vegetation growth increased significantly at a rate of 2.3 × 10^−3^ per year. However, the drivers of vegetation growth varied across different stages of the growing season. In the early growing season, an advanced SOS significantly promoted vegetation growth in forest and grassland, but this facilitative effect gradually diminished and turned inhibitory during the peak and late stages, during which warming became the primary driver of vegetation growth. Notably, the positive influence of SOS persisted until the fifth month of the growing season in forest but only until the fourth month in grassland. The results of this study supplement those of studies on vegetation growth in the CRCDR, elucidate the effects of the SOS on the dynamic process of vegetation growth, and offer insights into vegetation ecosystems.

## Graphical abstract

During the early growing season, the advanced SOS significantly promoted vegetation growth, but this promoting effect weakened and gradually shifted to a suppressive effect during the peak and late growing seasons. At the peak and late of the growing season, warming becomes the primary driver of increased vegetation growth.

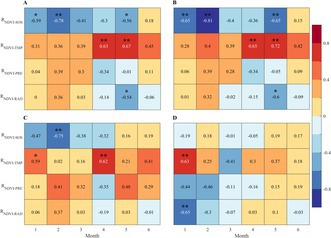

## 1 Introduction

Plant phenology refers to the sequential occurrence of developmental stages in plants that recur annually. It serves as a critical biological indicator for assessing ecosystem dynamics and their interactions with environmental conditions. Among these phenological events, spring phenology—particularly the start of the growing season (SOS)—is a key component, and understanding its variability is essential for evaluating vegetation responses to global climate change (Piao et al. 2019). In recent years, satellite‐derived normalized difference vegetation index (NDVI) data have become a widely used tool for examining SOS variations across diverse regions. The continuous, large‐scale, and frequent monitoring capabilities of satellite observations have profoundly improved our understanding of plant phenology (Gong et al. 2024; Piao et al. 2019; White et al. 2009). Numerous studies have found that SOS in the Northern Hemisphere has advanced due to climate warming (Barichivich et al. 2013; Piao et al. 2015; Schwartz et al. 2006; Su et al. 2022; Wang et al. 2023). However, while the SOS response to warming has significantly weakened in recent decades (Fu, Zhao, et al. 2015), the advancing SOS has affected the carbon, nitrogen, and water cycles in terrestrial ecosystems (Jiménez‐García et al. 2021; Piao et al. 2008). From a carbon balance perspective, earlier SOS extends the growing season, thereby enhancing vegetation growth (Myneni et al. 1997; Zhu et al. 2016) and increasing net carbon uptake by forests (Keenan et al. 2014). Furthermore, the advancement of SOS influences other ecological processes. For instance, it may heighten evapotranspiration, accelerating soil moisture depletion and reducing runoff (Chen et al. 2022; Lian et al. 2020), or increase nitrogen demand, thereby diminishing nitrogen use efficiency in forests (Elmore et al. 2016). Therefore, understanding the legacy effects of SOS on vegetation growth at different stages of the growing season is critical for quantifying the interplay between biotic and climatic factors in shaping vegetation dynamics.

Previous studies have demonstrated that changes in temperature and precipitation primarily regulate vegetation growth in the Northern Hemisphere; however, these effects exhibit regional variability (Guo et al. 2022; Keenan and Riley 2018; Piao et al. 2020). Moreover, recent research has reported the influence of SOS on spring and summer vegetation growth. For example, Zhou et al. found that SOS, rather than climatic factors, serves as the primary determinant of spring vegetation growth in temperate forests and grasslands in China (Zhao et al. 2021). Huang et al. observed that the advancement of SOS primarily reduced spring vegetation growth in the Northern Hemisphere but increased summer vegetation growth. However, this relationship is expected to reverse in the future (Huang et al. 2024). Currently, research has only examined the legacy effects of SOS on vegetation growth during spring and summer, and the interconnections among SOS, climate change, and vegetation growth across different growing season phases are not fully understood. Therefore, it is imperative to investigate the interactive effects of SOS and climatic factors on vegetation growth across different stages of the growing season. Such efforts are crucial for advancing our understanding of vegetation dynamics and improving predictions of climate change impacts on ecosystems.

In this study, we utilized satellite‐derived NDVI data (2001–2020) to investigate vegetation growth dynamics in the CRCDR. We also explored how the SOS and climatic factors (i.e., temperature, precipitation, radiation) influence vegetation growth dynamics during the growing season. Our analysis aimed to address the following three key questions: (1) What are the dominant factors in vegetation growth in the CRCDR in different months of the growing season? (2) How does the SOS affect vegetation growth in different months of the growing season? and (3) Do the dominant factors in vegetation growth vary by vegetation type?

## 2 Materials and Methods

### 2.1 Study Area

**FIGURE 1:**
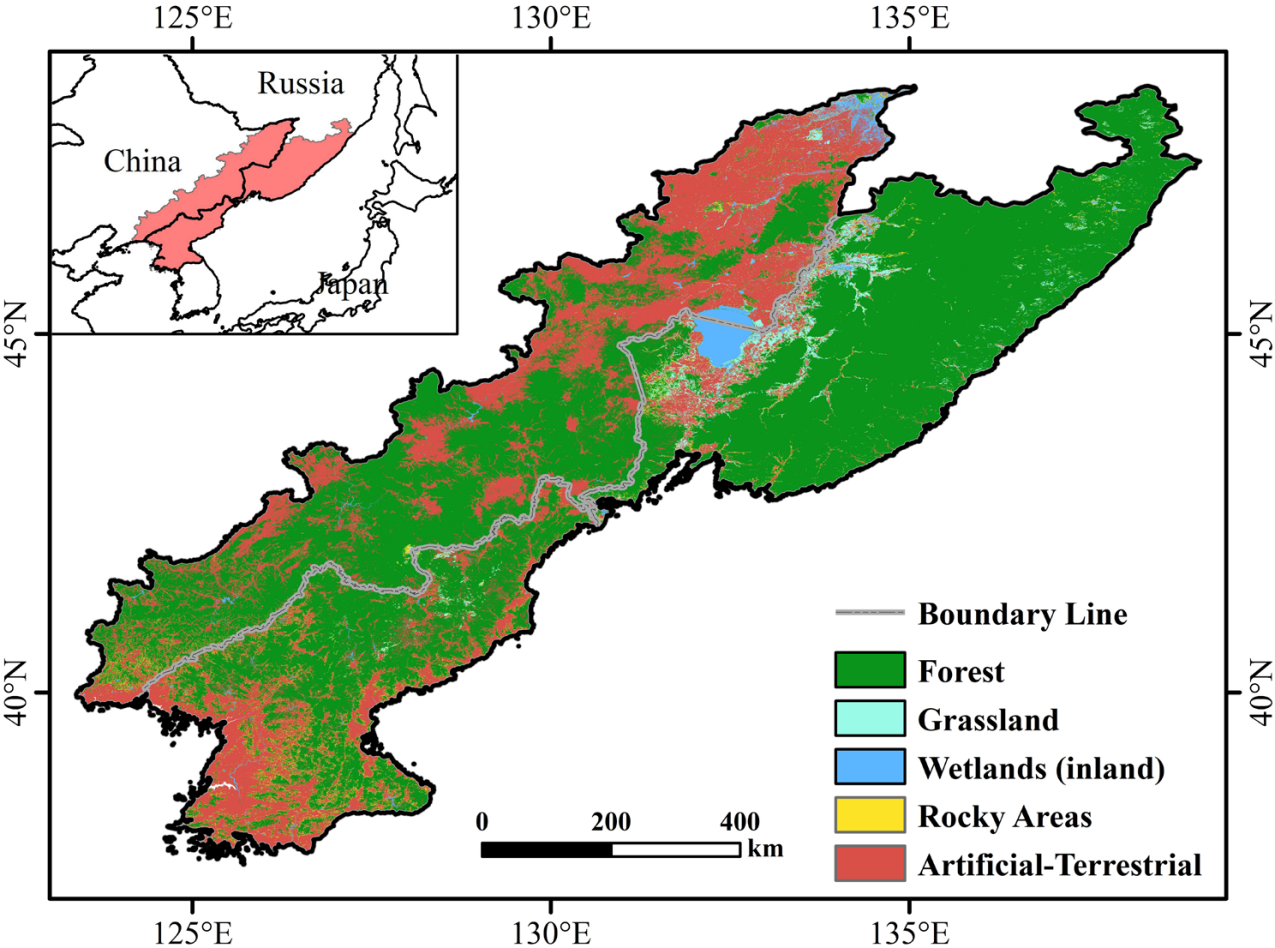
Location and natural vegetation distribution of the CRCDR.

The CRCDR is located in Northeast Asia, centered in the Changbai Mountains, with longitude and latitude of 123°37′ E–139°00′ E, 37°58′ N–48°45′ N (Figure 1). It includes parts of Heilongjiang, Jilin, and Liaoning provinces of China, the whole territory of DPRK, and Primorsky Krai in Russia. The climate in the study area is a temperate continental monsoon climate, with an annual mean temperature of approximately 3.96 C and an annual precipitation of about 787.4 mm. The overall topography of the CRCDR is dominated by mountainous terrain, with a forest coverage of up to 70%, making it an important ecological security barrier and ecologically fragile zone in Asia (Liu et al. 2021; Qian et al. 2003).

### 2.2 Data Sources

#### 2.2.1 Vegetation Growth Proxy

In this study, the satellite‐derived vegetation index NDVI is used as an indicator of vegetation growth. NDVI has long been regarded as a reliable metric for assessing vegetation health and is widely applied in ecological research (Zeng et al. 2022). The NDVI was sourced from the MOD13A1 dataset, with a temporal resolution of 16 days, a spatial resolution of 500 m, and a time span of January 2001 to December 2020.

#### 2.2.2 Climate Dataset

The climate data were cited from the TerraClimate monthly climate dataset (http://www.climatologylab.org) with a spatial resolution of 4 km. The TerraClimate dataset is a reanalysis dataset of monthly climate and climatic water balance on the global terrestrial surface that is very useful in global scale ecological and hydrological studies. We acquired related climatic data (i.e., monthly average maximum and minimum temperatures, monthly cumulative precipitation, and monthly cumulative radiation) for the period of January 2001 to December 2020 to characterize the climatic conditions of the CRCDR. We used the mean of the monthly average maximum and minimum temperatures in the TerraClimate dataset as the monthly average temperature of the CRCDR.

#### 2.2.3 Snow Cover Dataset

We utilized the daily Normalized Difference Snow Index (NDSI) derived from the MOD10A1 dataset to determine the snow cover period of each pixel in the CRCDR from January 2001 to December 2020. The spatial resolution of the dataset was 500 m, and its temporal resolution was 1 day.

#### 2.2.4 Vegetation Distribution Dataset

To characterize the land cover of CRCDR, we utilized the global terrestrial habitat map produced by Jung et al. (2020), which displays the distribution of the International Union for Conservation of Nature (IUCN) habitat categories (Level 1) at a 100‐m resolution. Terrestrial biomes in the CRCDR consist of forest, grassland, wetlands, and artificial‐terrestrial (Figure 1).

### 2.3 Methodology

#### 2.3.1 Data Pre‐Processing

The CRCDR has a high latitude and a long snow cover period. However, recent studies have highlighted that snow cover during the non‐growing season can distort NDVI values, leading to biased vegetation phenology identification (Piao, Wang, et al. 2011; Shen et al. 2013; Wang et al. 2013; Wang et al. 2018; Yu et al. 2010). To mitigate this influence, we used the daily NDSI to identify snow cover dates and set 0 as the threshold for snow detection (Xiaoyan et al. 2024). Subsequently, we generated a high‐quality NDVI dataset, relying on the pixel reliability data layer from MOD13A1. Any outliers exceeding three times the standard deviation of this NDVI dataset during the annual snow cover period were eliminated. Finally, we designated the minimum NDVI value from this dataset as the background value for the non‐growing season of vegetation. We also removed pixel points with annual average NDVI values less than 0.1, which usually indicated sparsely vegetated or bare areas.

To exclude the impact of anthropogenic activities on vegetation cover, we did not consider farmland. For ease of computation, we resampled all the data to a spatial resolution of 4 km.

#### 2.3.2 Extraction of Vegetation Phenology

To eliminate the influence of outliers, we first used the iterative Savitzky–Golay filtering method to smooth the snow‐free NDVI dataset (Figure S13). Using the Polyfit‐Maximum method (Piao et al. 2006), we extracted the SOS of the CRCDR for the period 2001–2020. The Savitzky–Golay filtering method reflects vegetation phenology well and has been widely used to extract phenological periods in other regions (Bao et al. 2021; Fu et al. 2014; Piao et al. 2015; Shen et al. 2019; Wang et al. 2016), and unlike other methods, this method requires no manual setting of initial parameter values and parameter value ranges, making it less susceptible to human influence. Then, we performed polynomial fitting on the NDVI to obtain daily time series, with the number of polynomial fits set to 6 (Piao et al. 2006) in most studies (Equation 1). We calculated the rate of change of the fitted NDVI and designated the time corresponding to the maximum rate of change as the date of SOS (Equation 2).

(1) $$\text{NDVI}\left(t\right)=\alpha_{0}+\alpha_{1}t^{1}+\alpha_{2}t^{2}+\ldots +\alpha_{6}t^{6}$$

(2) $$\begin{array}{c} {\text{NDVI}}_{\text{ratio}}\left(t\right)=\frac{\text{NDVI}\left(t+1\right)-\text{NDVI}\left(t\right)}{\text{NDVI}\left(t\right)} \end{array}$$

where *t* denotes the day of year (DOY), $\text{NDVI}\left(t\right)$ denotes the NDVI value of the *t*th day, and $\alpha_{0}$, …, $\alpha_{6}$ denote the fitting coefficients determined by the least squares regression method. The time *t* value was set when the rate of change was maximized as the result for seeking the SOS outcome.

#### 2.3.3 Analyses of Vegetation Growth of the Growing Season and Their Dependencies on SOS and Climate Factors

After calculation, it is determined that in over 95% of the study area, the vegetation growing season lasts for 5–7 months, with an average length of 179 days. Therefore, we focus solely on the vegetation dynamics occurring from the first month to the sixth month of the growing season. In this study, the NDVI data was used also for determining the vegetation growth from the first to the sixth month of the growing season. The average monthly NDVI in the current month of the SOS was defined as the vegetation growth in the first month of the growing season, and so on, thus defining the vegetation growth in the consecutive 6 months of the growing season (Figure S6).

In this study, the temporal trends and their significance of SOS, vegetation growth proxy (i.e., NDVI) and climate data were analyzed using the Theil‐Sen estimator and the Mann‐Kendal statistical test. This method is commonly applied in the trend analysis of long time‐series data (Liu et al. 2019; Yuan et al. 2013; Zhao et al. 2021). We applied pearson's partial correlation analysis to quantify the relative importance of SOS days and climate factors (mean temperature, precipitation sum, insolation sum) during the growing season on vegetation growth. In addition, the maximum correlation coefficient between vegetation growth and potential factors within the same pixel was calculated to determine the dominant role of a specific factor in vegetation growth. This method has been successfully and widely applied in previous studies to eliminate the confounding effects of correlations among multiple variables (Fu, Piao, et al. 2015; Li, Fu, et al. 2021; Li, Wang, et al. 2021; Sun et al. 1998).

## 3 Results

### 3.1 Spatiotemporal Pattern of SOS in the CRCDR

**FIGURE 2:**
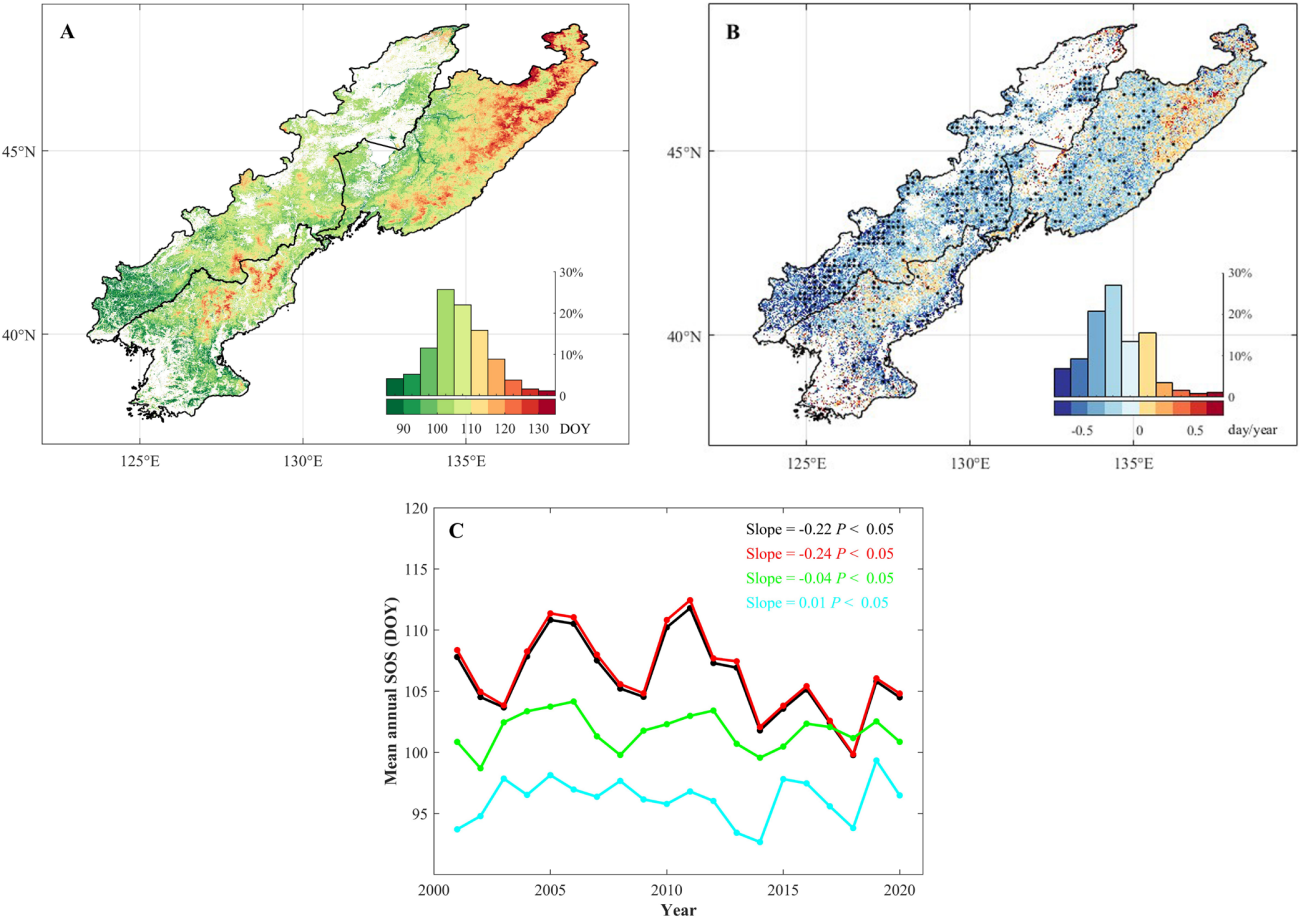
Spatiotemporal distribution of SOS in the CRCDR from 2001 to 2020. (A) Spatial distribution of average value of SOS; (B) spatial distribution of SOS trend; (C) temporal change in the average value of SOS. The histogram indicates the statistics of the trend value denoted by each color. Black dots denote the pixel points with statistically significant phenological trends when *p* < 0.05. The black curve denotes the entire CRCDR. The red curve denotes the forest subregion, the green curve denotes the grassland subregion, and the cyan curve denotes the wetlands subregion.

The average SOS across the CRCDR was 106 ± 9 day of year (DOY). Spatially, later SOS were concentrated in the higher‐elevation mountainous areas of the northern region. Sen trend analysis showed that from 2001 to 2020, the overall SOS advanced by 0.22 days per year (*p* < 0.05). Among all CRCDR pixels, 77.54% exhibited an advancing SOS trend, with 19.41% showing a significant advance. Notably, the SOS in Liaoning Province of China and the northern coastal areas of the DPRK advanced significantly, typically by 0.45–0.75 days per year. In contrast, delayed SOS trends were primarily located in high‐elevation parts of the Changbai and Sikhote‐Alin Mountains (Figure 2B). Forest SOS mainly ranged from 103 to 110 DOY and advanced at a relatively faster rate (Figure 2C). Grassland advanced by 0.04 days per year, whereas wetlands showed a slight delay of 0.01 days per year.

### 3.2 Spatiotemporal Distribution of Vegetation Growth

**FIGURE 3:**
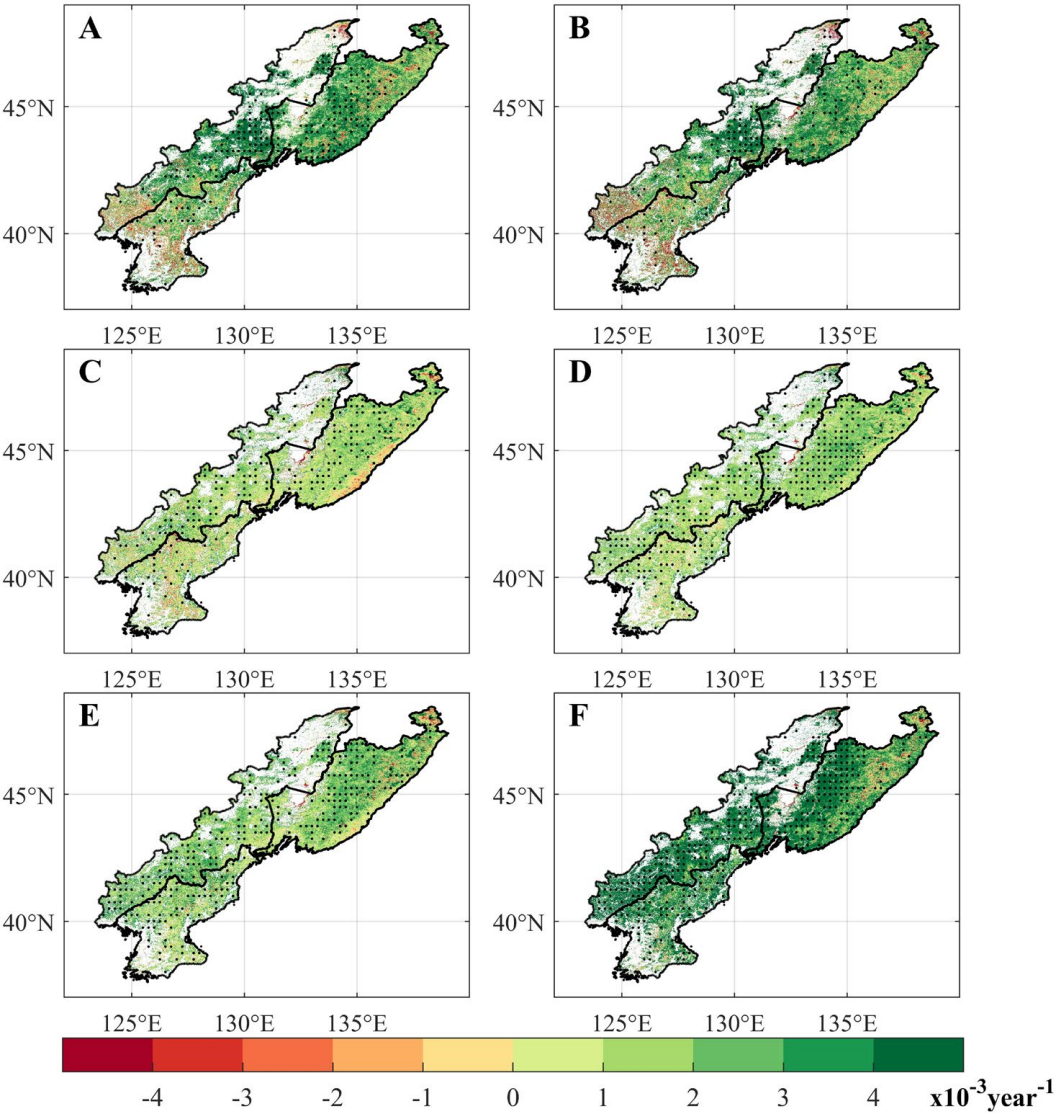
Spatiotemporal distribution of NDVI trend in the CRCDR from 2001 to 2020. (A–F) Denote the first to sixth months of the growing season. Black dots denote the pixel points with statistically significant NDVI trends when *p* < 0.05.

Overall, vegetation growth of the CRCDR exhibited an upward trend across all months of the growing season, although the rate of increase displayed spatial heterogeneity. In the first month of the growing season, NDVI rose in 82.7% of the region, primarily in the central and eastern parts of the CRCDR, while a slight decline occurred in the southwest (Figure 3A). In the second month of the growing season, the spatial pattern of NDVI trends remained similar (Figure 3B). During the third, fourth, and fifth months, NDVI increased in 82.4%, 90.9%, and 89.4% of the CRCDR, respectively (Figure 3C–E). However, in the sixth month of the growing season, a more extensive increase in NDVI (covering approximately 92.6% of the CRCDR) was observed (Figure 3F).

**FIGURE 4:**
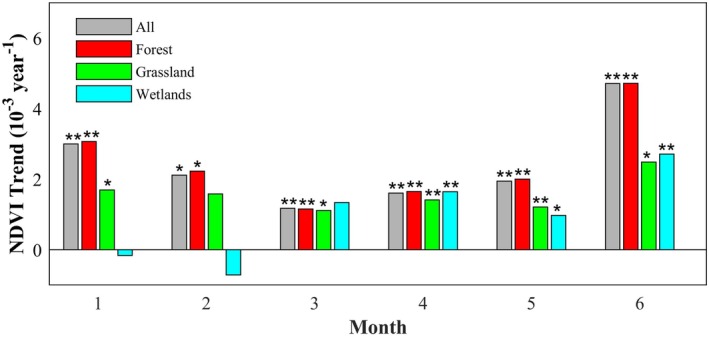
Trend of NDVI values in different CRCDR subregions during the growing season from 2001 to 2020. “*” means that the results are statistically significant (*p* < 0.05), and “**” means that the results are statistically extremely significant (*p* < 0.01).

From 2001 to 2020, the NDVI of the CRCDR during the growing season increased at a rate of 2.3 × 10^−3^ per year (*p* < 0.05), though the growth rate varied by month and vegetation type (Figure 4). Specifically, during the first to sixth months of the growing season, the NDVI growth rate initially slowed, with the slowest increase occurring in the third month (1.17 × 10^−3^ per year, *p* < 0.01). Subsequently, the growth rate gradually accelerated, reaching its peak in the sixth month (4.71 × 10^−3^ per year, *p* < 0.01). Across different vegetation types, forest, with more complex canopy structures, showed higher NDVI growth rates than grassland and wetlands in most months of the growing season. Grassland showed relatively stable growth rates throughout the growing season. Interestingly, during the first 2 months of the growing season, wetlands NDVI decreased at rates of 0.16 × 10^−3^ per year (*p* > 0.05) and 0.71 × 10^−3^ per year (*p* > 0.05), respectively.

### 3.3 Spatiotemporal Pattern of Climate Conditions in the CRCDR

**FIGURE 5:**
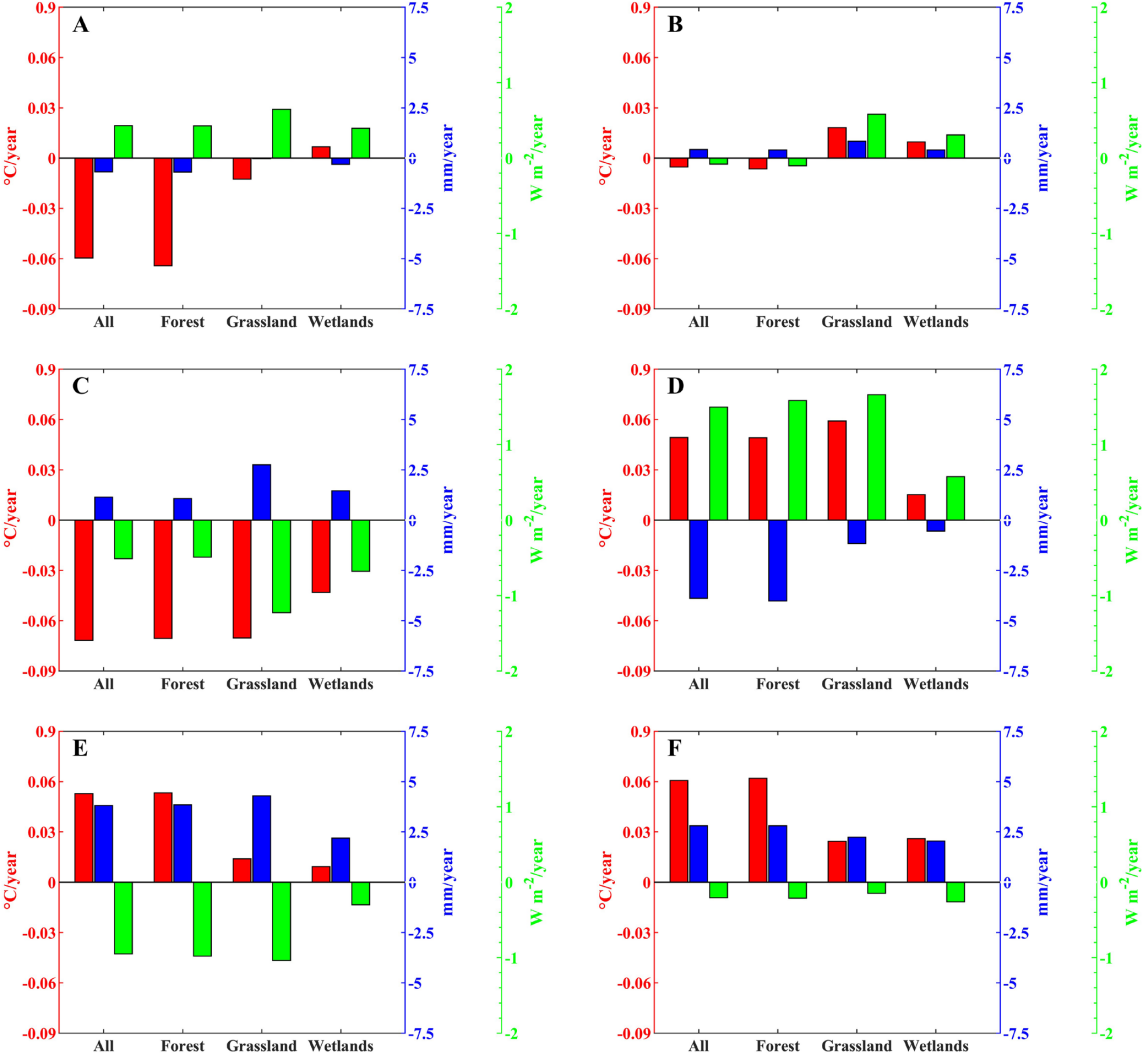
Histogram of climate change trend of the CRCDR in the growing season from 2001 to 2020. (A–F) Denote the first to sixth months of the growing season. The red bars denote temperature, the blue bars denote precipitation, and the green bars denote radiation.

To understand the climate characteristics of the CRCDR region, we analyzed the trends in temperature, precipitation, and solar radiation from 2001 to 2020. The results revealed spatially varying trends in climate conditions across different months of the growing season (Figure 5, Figures S3–S5). Specifically, during the first 3 months of the growing season, temperatures showed a decreasing trend. However, in the fourth month, 90.5% of the region experienced significant warming (Figure S3), with a warming rate of 0.049°C per year, and this significant warming trend continued into the sixth month, reaching a rate of 0.06°C per year. Precipitation trends were more complex, with slower changes during the first 3 months of the growing season and more rapid changes in the latter 3 months (Figure 5). In the fourth month, 80.7% of the region experienced a significant decrease in precipitation, with a reduction rate of −3.89 mm per year. In contrast, precipitation increased in most of the CRCDR region during the last 2 months of the growing season. Furthermore, the radiation trend was opposite to that of precipitation.

### 3.4 Response of Vegetation Growth to the SOS and Climate Factors

**FIGURE 6:**
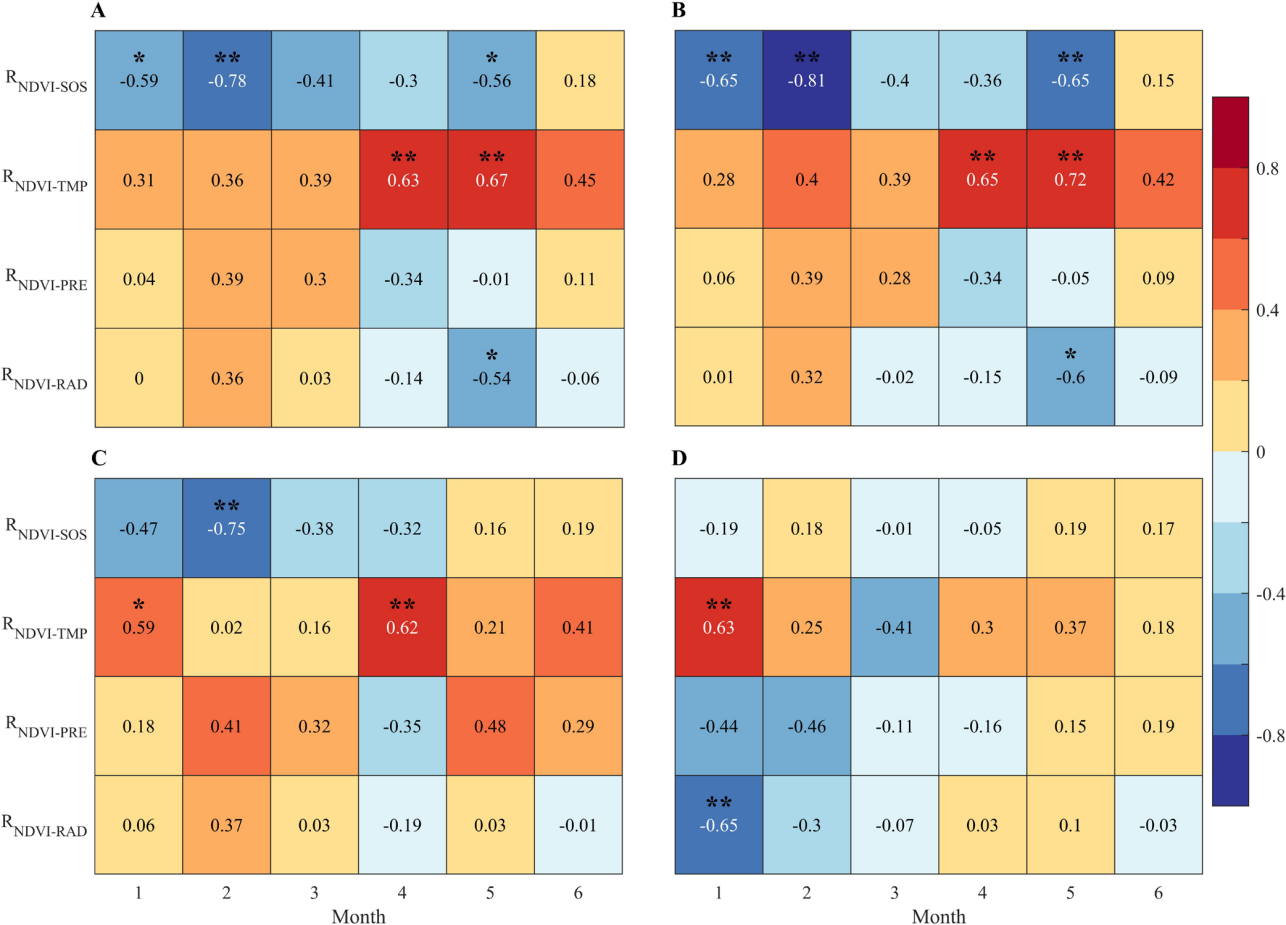
Correlation coefficients between NDVI and factors in the growing season in different CRCDR subregions. (A–D) Denote the entire CRCDR, forest, grassland, and wetlands subregions. “*” means that the results are statistically significant (*p* < 0.05), and “**” means that the results are statistically extremely significant (*p* < 0.01).

To investigate the effects of SOS and climate factors on vegetation growth in the CRCDR, we calculated the partial correlation between NDVI and factors from 2001 to 2020. The results showed that for forests, during the first 2 months of the growing season, NDVI was significantly negatively correlated with SOS (*p* < 0.01) and weakly positively correlated with temperature. The negative correlation between NDVI and SOS was strongest in the second month (Figure 6, Figures S7 and S8). From the third to the fifth month of the growing season, NDVI remained negatively correlated with SOS in most areas, although the spatial extent of this relationship gradually shrank, while areas positively correlated with temperature increased (Figure 6, Figures S9–S11). In the sixth month, NDVI showed positive correlations with both SOS and temperature, with the correlation with temperature being stronger (Figure 6, Figure S12). Notably, in forests, the promoting effect of SOS can persist until the fifth month of the growing season, whereas in grasslands, this effect only lasts until the fourth month. Additionally, precipitation had a stronger positive correlation with grassland NDVI. For wetlands, in the first 2 months of the growing season, temperature and radiation were positively and negatively correlated with wetland NDVI, respectively, with the relationship being significant in the first month (*p* < 0.01), while the relationship between SOS and wetland NDVI was weak (Figure 6).

## 4 Discussion

### 4.1 Relationship of Vegetation Growth Between Climate Factor and SOS

**FIGURE 7:**
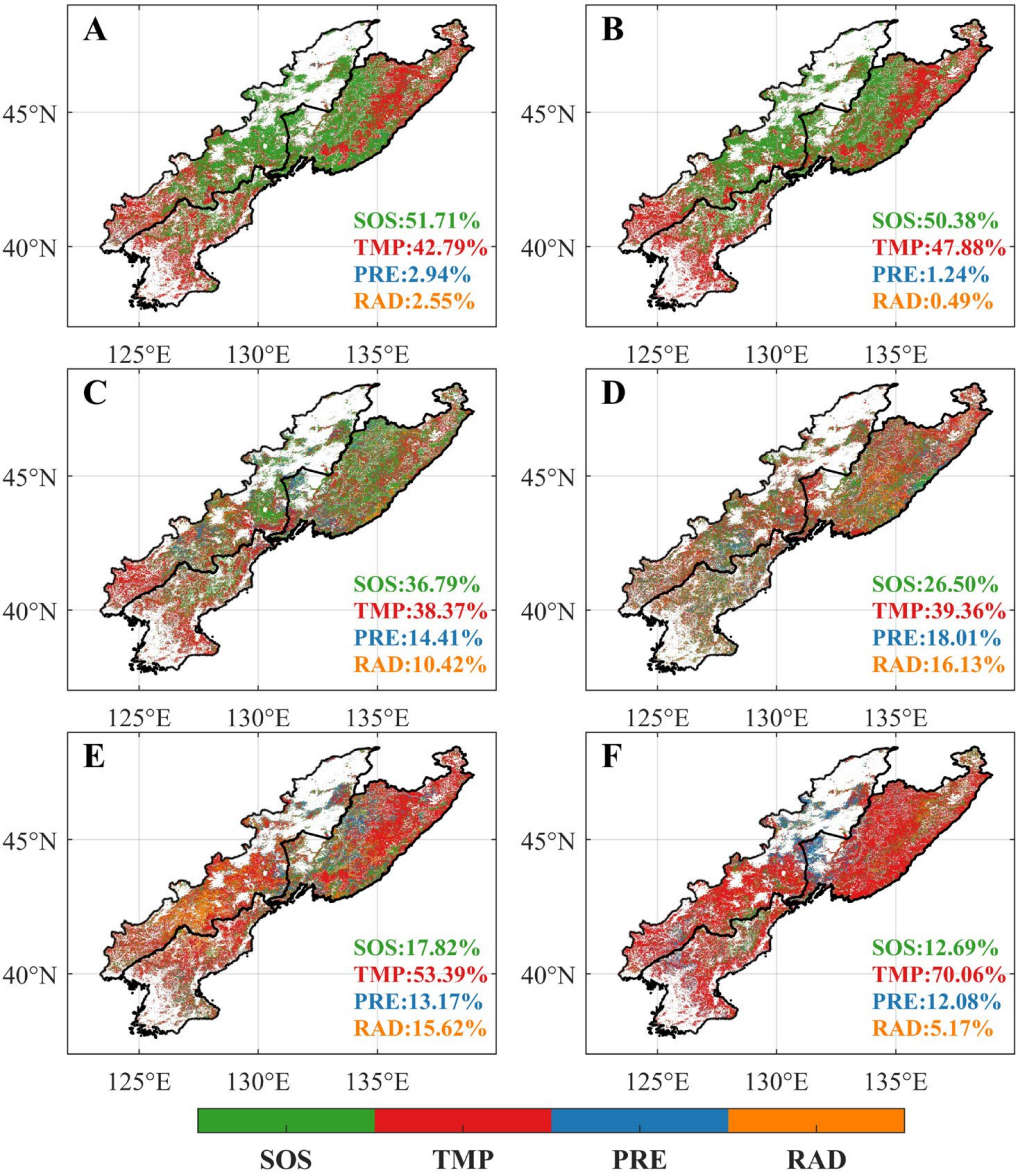
Distribution of the maximum correlation coefficients between vegetation growth and SOS, temperature, precipitation, and radiation (A–F) denote the first to sixth months of the growing season.

Previous studies have reported residual effects of SOS, which may either enhance or reduce vegetation growth (Piao, Cui, et al. 2011; Wang et al. 2011). Our results indicate that during the early growing season, specifically the first 2 months (Figures S1 and S2), advanced SOS stimulated vegetation growth across much of the CRCDR region (Figure 7), which was closely associated with the earlier onset of photosynthesis and development (Keenan et al. 2014). Interestingly, although temperature controlled vegetation growth in some areas, the unusually cold spring temperatures in this region may have suppressed vegetation growth to some extent by inhibiting the activity of photosynthetic enzymes involved in carbon assimilation (Moore et al. 2021; Shi et al. 2014). This climatic phenomenon could be linked to anomalies in atmospheric oscillations (Fu et al. 2023; Kim et al. 2023). In the subsequent 3 months, vegetation growth entered the peak growing season (Figure S1), where the promoting effect of the advanced SOS on vegetation growth weakened, and warming gradually became the dominant factor influencing vegetation growth (Figure 7). However, considering that vegetation growth was nearing its peak, the combined effects of SOS advancement and warming were less pronounced than in the previous stage. Notably, due to anomalous sea surface temperatures in the North Atlantic, the third month of the growing season experienced the most significant cooling event (Li et al. 2024), which counteracted the positive effect of the advanced SOS and may be one of the reasons for the lower vegetation growth during this period. The relationship between SOS and vegetation growth seems to change during the late growing season. This shift may be due to changes in the plant life cycle, as the advanced SOS dominated changes in the growing season peak, which could indicate a faster decline in photosynthetic capacity (Gonsamo et al. 2018; Huang et al. 2023), although this had a limited effect on vegetation growth in the region. Conversely, warming at the late growing season may positively influence vegetation growth by slowing the speed of chlorophyll degradation during autumn leaf senescence (Fracheboud et al. 2009; Liu et al. 2016), counteracting the negative effects of the advanced SOS. It appears that precipitation and radiation are not the primary factors affecting vegetation growth in the CRCDR region, likely because the area enjoys relatively abundant moisture and sunlight, preventing them from becoming a stressor for vegetation growth. Furthermore, the high forest cover in the CRCDR contributes to this stability; forests can tap into deep soil moisture through their extensive root systems to buffer against fluctuations in precipitation (Liu et al. 2018). In addition, the complex canopy structure of forests helps mitigate the effects of broader climatic variability (Thom et al. 2020; Verheyen et al. 2024).

For different vegetation types, grassland shows a shorter duration of growth promotion from SOS compared to forest, and is more sensitive to precipitation (Figure 6). This may be due to the fact that grassland is primarily composed of herbaceous plants, which have shorter lifecycles and shallower root systems, limiting their ability to absorb water from deeper soil layers like trees in forest. Therefore, grassland plants rely more on surface soil moisture (Liu et al. 2024). For wetlands, the relatively stable SOS has a limited impact on vegetation growth; however, temperature increases more strongly promote early vegetation growth in wetlands. Despite this, the rise in wetland temperature is not substantial, and higher solar radiation may accelerate the evaporation of soil moisture in the early growing season (Wang et al. 2024), leading to a decrease in soil moisture and potentially causing short‐term drought stress, which in turn may suppress wetlands vegetation growth (Figures 2, 4, and 6).

### 4.2 Limitations and Future Studies

In this study, we used the MOD13A1 NDVI data product with a 500 m resolution to extract the pixel‐scale SOS of the CRCDR over the last two decades. Because of the special nature of the bordering areas, we could not obtain effective in situ phonological data, making it impossible to verify the accuracy of the SOS assessed by remote‐sensing data for the time being. We believe that as more phenology data are acquired, the remote‐sensing inversion data will be verified and corrected by building phenology observation stations in the CRCDR or using manually collected measured data. Moreover, the CRCDR has abundant vegetation types, covering almost all vegetation types available from middle latitudes to polar regions (Zhang 2020). Future studies should investigate the differentiated effects of the phenology of diverse vegetation types on vegetation growth to fill the gap of related studies about the CRCDR and provide useful reference information for sustainable development of the CRCDR.

## 5 Conclusions

This study analyzed the effects of SOS and climatic factors on vegetation growth during the growing season in the CRCDR region from 2001 to 2020. We found that the SOS in the CRCDR advanced at a rate of 0.22 days per year. NDVI, used as a proxy for vegetation productivity, increased significantly at a rate of 2.3 × 10^−3^ per year. However, the factors driving vegetation growth varied across different stages of the growing season. The advanced SOS significantly promoted vegetation growth in forest and grassland during the early growing season, but this facilitative effect gradually weakened and turned inhibitory during the peak and late stages. The positive influence of SOS extended to the fifth month of the growing season in forest but only to the fourth month in grassland. As the growing season progressed, temperature gradually replaced SOS as the primary driver of vegetation growth. This study emphasizes the importance of spring phenology changes in modeling vegetation growth processes, providing insights into vegetation growth under future climate conditions.

## Author Contributions

**Lujie Zhao:** conceptualization (lead), formal analysis (lead), writing – original draft (lead). **Jihao Zhang:** conceptualization (equal), data curation (equal), writing – review and editing (equal). **Duqi Liu:** data curation (equal), software (equal), writing – review and editing (equal). **Xiao Huang:** funding acquisition (equal), investigation (equal), resources (equal), writing – review and editing (equal). **Zhen Xu:** funding acquisition (equal), investigation (equal), resources (equal), writing – review and editing (equal). **Guishan Cui:** investigation (lead), project administration (lead), resources (lead).

## Conflicts of Interest

The authors declare no conflicts of interest.

## Supporting information


Data S1.

## Data Availability

The MODIS NDVI data are obtained from the Goddard Space Flight Center (https://ladsweb.modaps.eosdis.nasa.gov/), and the climate data used in this study are downloaded from the climatology lab (http://www.climatologylab.org/terraclimate.html). The snow cover data are obtained from MOD10A1 provided by the National Snow and Ice Data Center (https://nsidc.org/data/mod10a1/versions/61). The maps of terrestrial habitat types are available at https://doi.org/10.5281/zenodo.3666245.
