# Supplementary material for: As the Growing Season Progresses, the Key Driving Factor of Vegetation Growth Shifts From Spring Phenology to Temperature in the Cross‐Border‐Region of Northeast Asia
Source: Ecol Evol. 2025 May 8;15(5):e71384. doi: 10.1002/ece3.71384 (PMC12059557; doi:10.1002/ece3.71384)
Supplement: Supplementary file 1 — Data S1. [file ECE3-15-e71384-s001.docx]

**Supplementary file for**

**As the Growing Season Progresses, the Key Driving Factor of Vegetation Growth Shifts from Spring Phenology to Temperature in the Cross-border-Region of Northeast Asia**

Lujie Zhao^a^, Jihao Zhang^b^, Xiao Huang^b^, Duqi Liu^b^, Zhen Xu^b^, Guishan Cuia,^b,*^

^a^ College of Integration Science, Yanbian University, Yanji, 133002, China

^b^ College of Geography and Ocean Sciences, Yanbian University, Yanji, 133002, China

^*^ Corresponding author: Guishan Cui, e-mail: cuiguishan@ybu.edu.cn, tel: +86-138-4338-1515.

Lujie Zhao and Jihao Zhang should be considered joint first author.


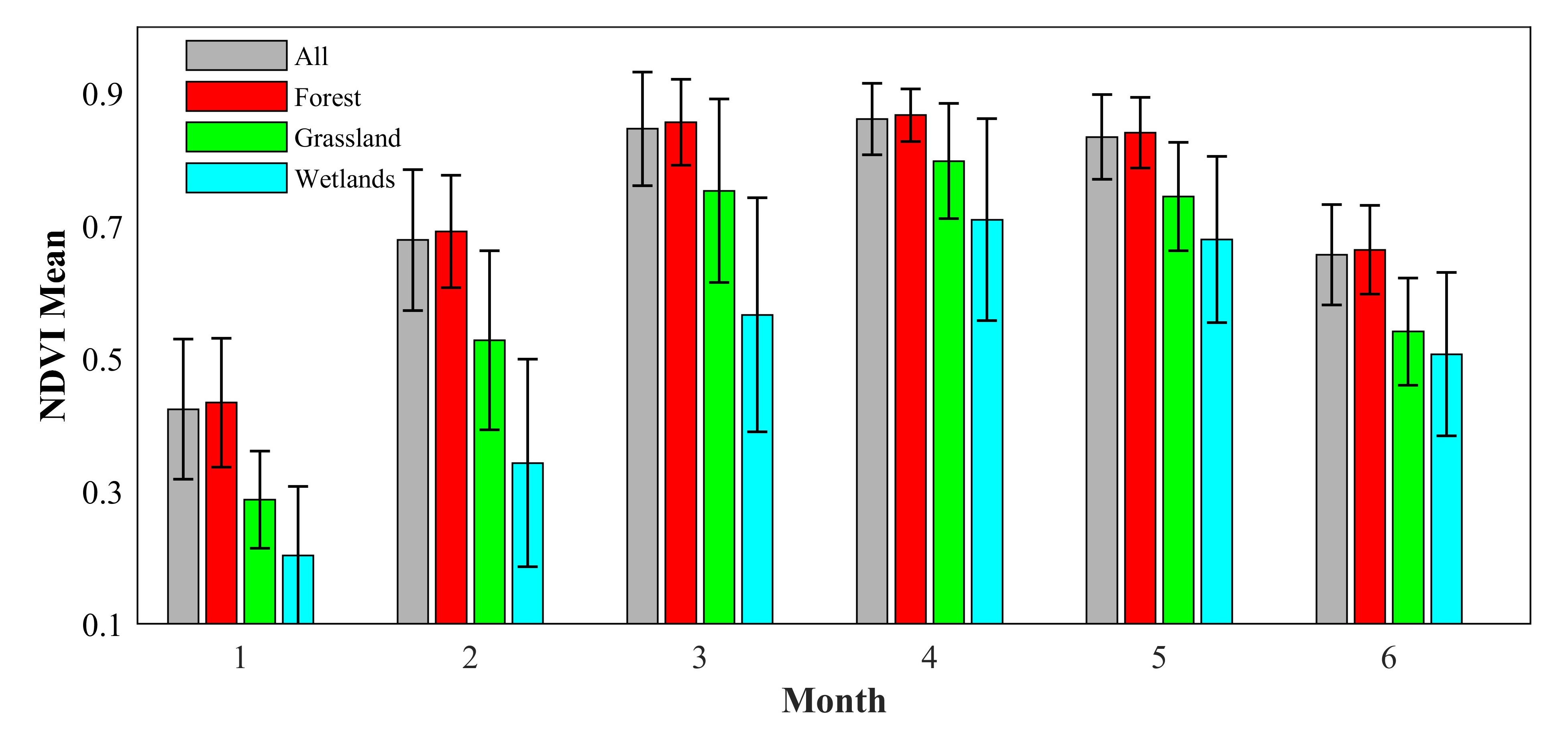


**Supplementary Figure 1.** NDVI mean value in different CRCDR subregions during the growing season from 2001 to 2020.


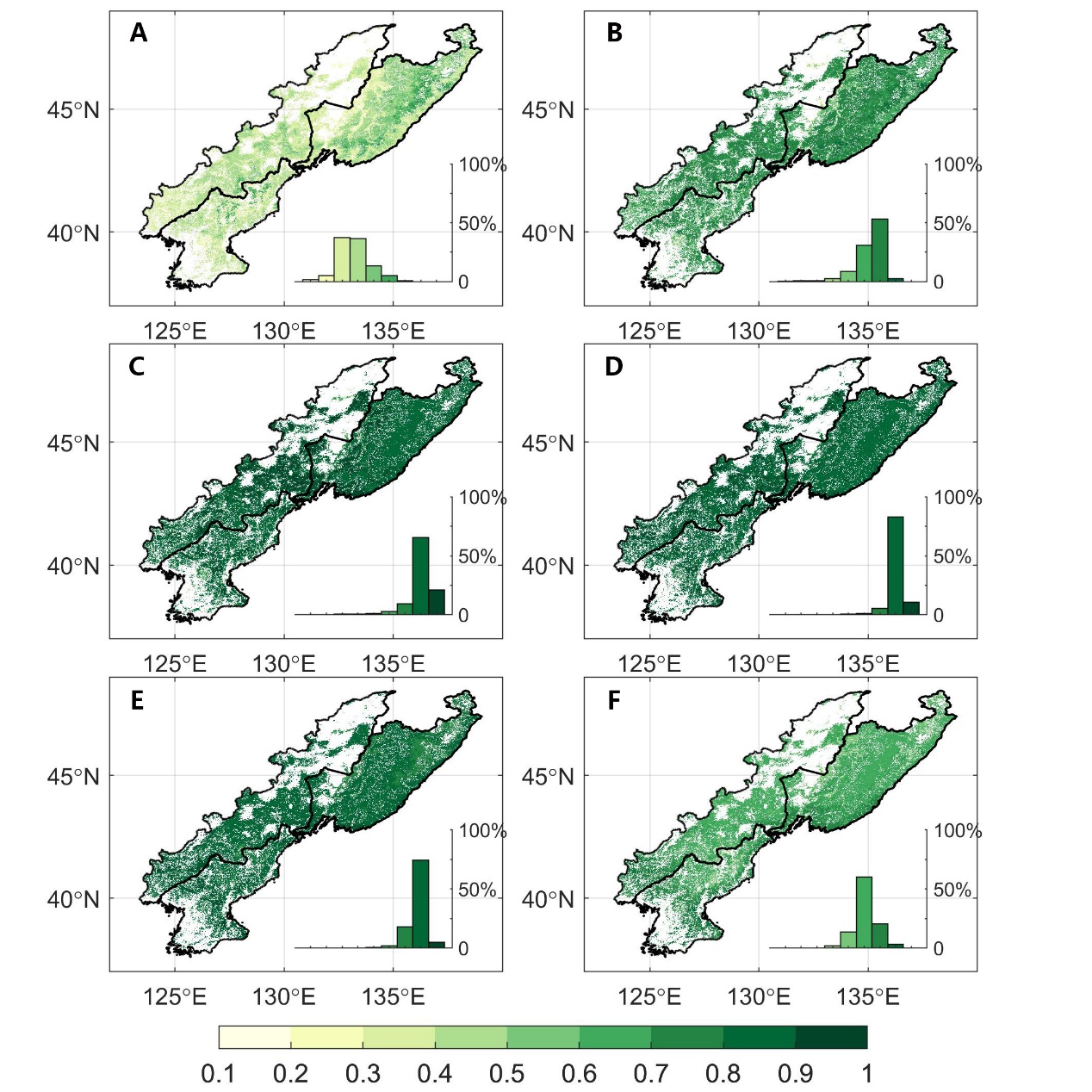


**Supplementary Figure 2.** Spatial distribution of mean NDVI value in the CRCDR from 2001 to 2020. (A-F) denote the first to sixth months of the growing season.


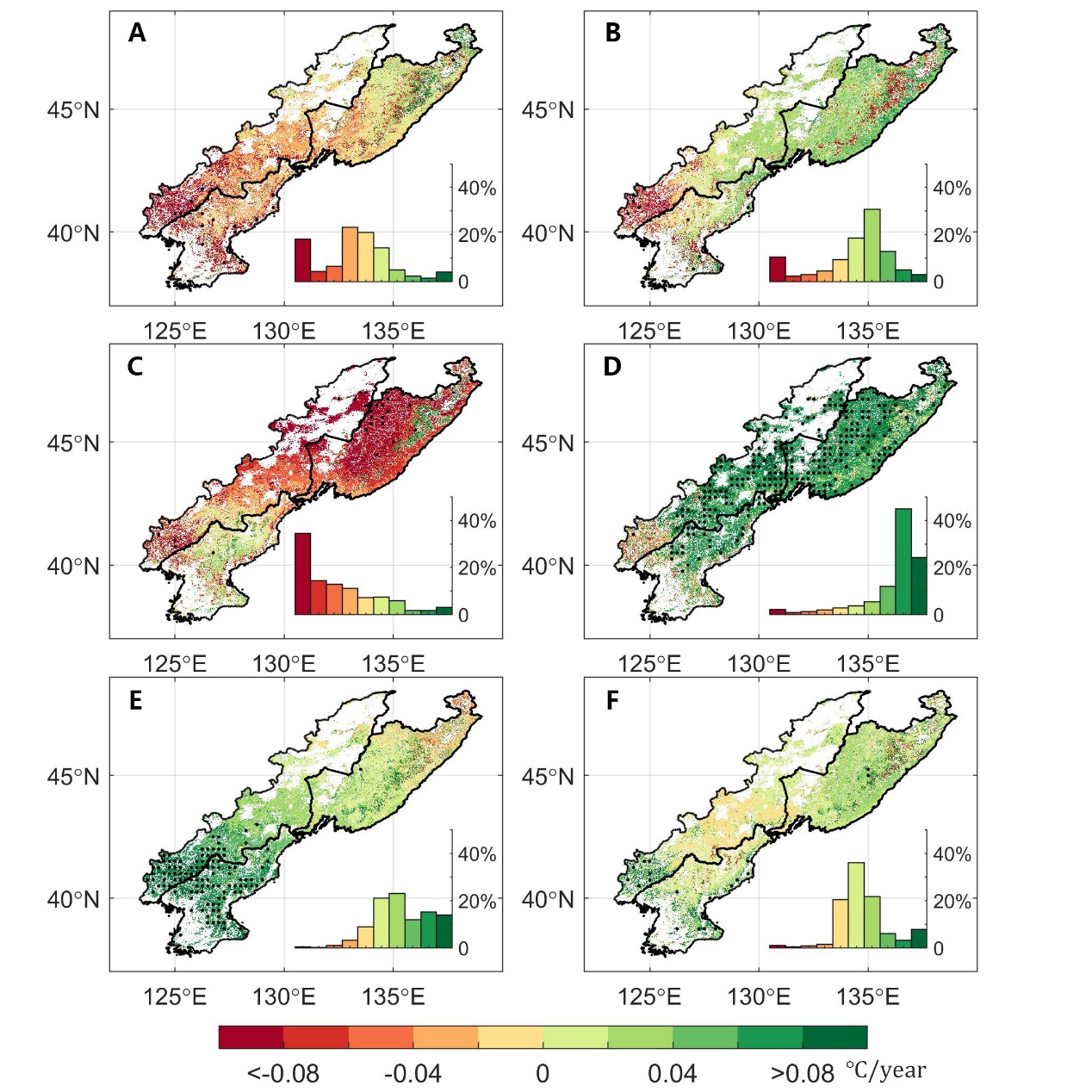


**Supplementary Figure 3.** Spatial distribution of tmean trend in the CRCDR from 2001 to 2020. (A-F) denote the first to sixth months of the growing season. Black dots denote the pixel points with statistically significant trends when p < 0.05.


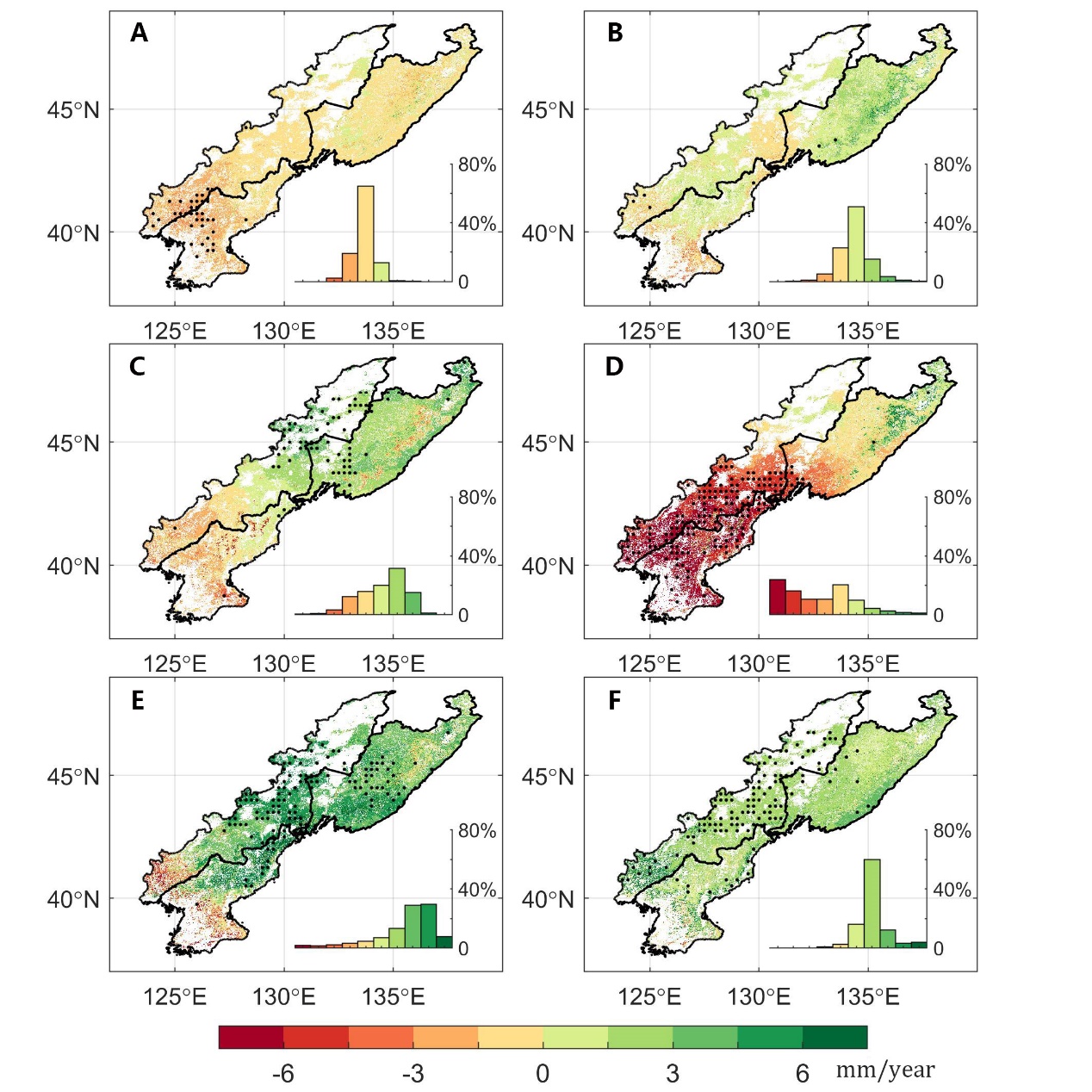


**Supplementary Figure 4.** Spatial distribution of precipitation trend in the CRCDR from 2001 to 2020. (A-F) denote the first to sixth months of the growing season. Black dots denote the pixel points with statistically significant trends when p < 0.05.


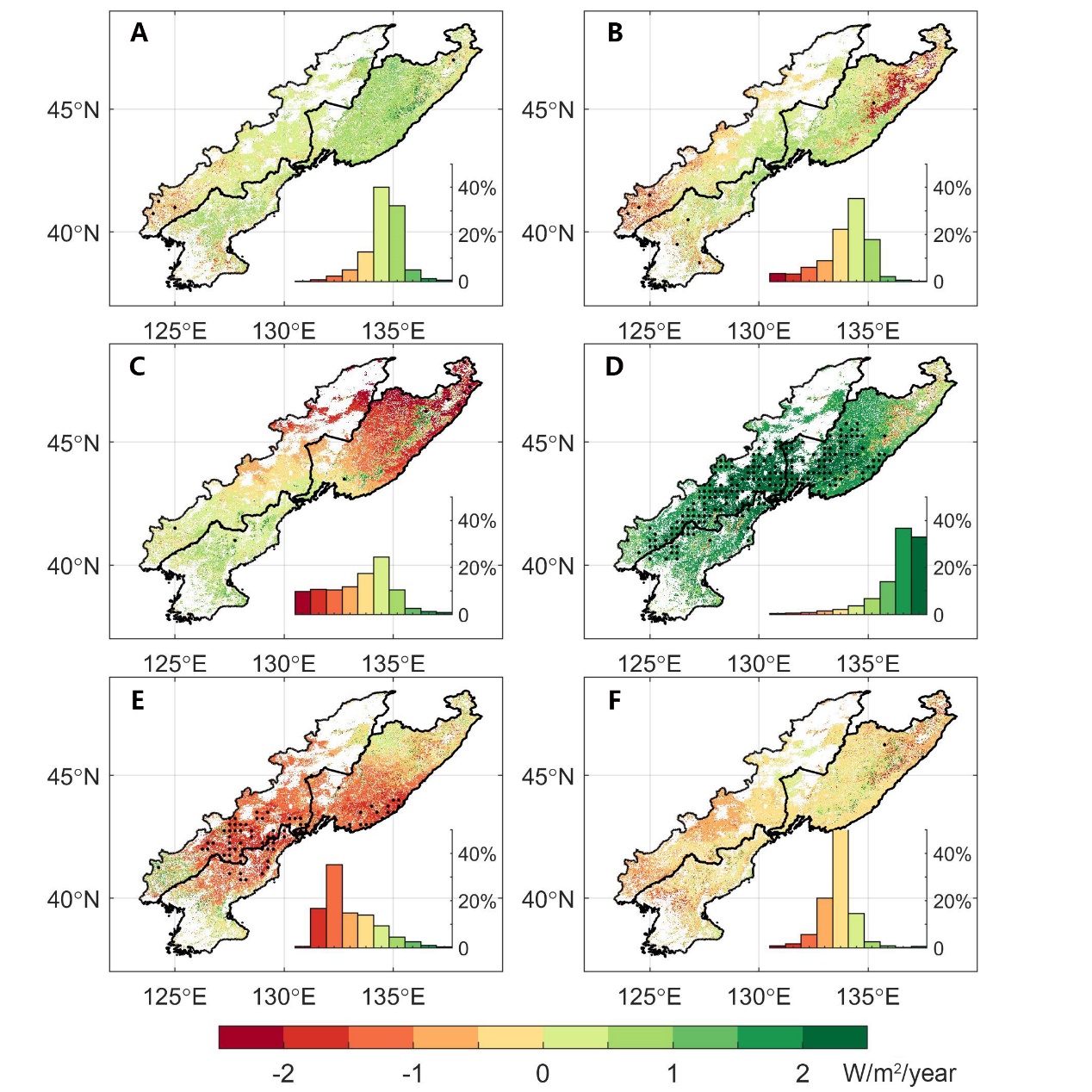


**Supplementary Figure 5.** Spatial distribution of radiation trend in the CRCDR from 2001 to 2020. (A-F) denote the first to sixth months of the growing season. Black dots denote the pixel points with statistically significant trends when p < 0.05.

**
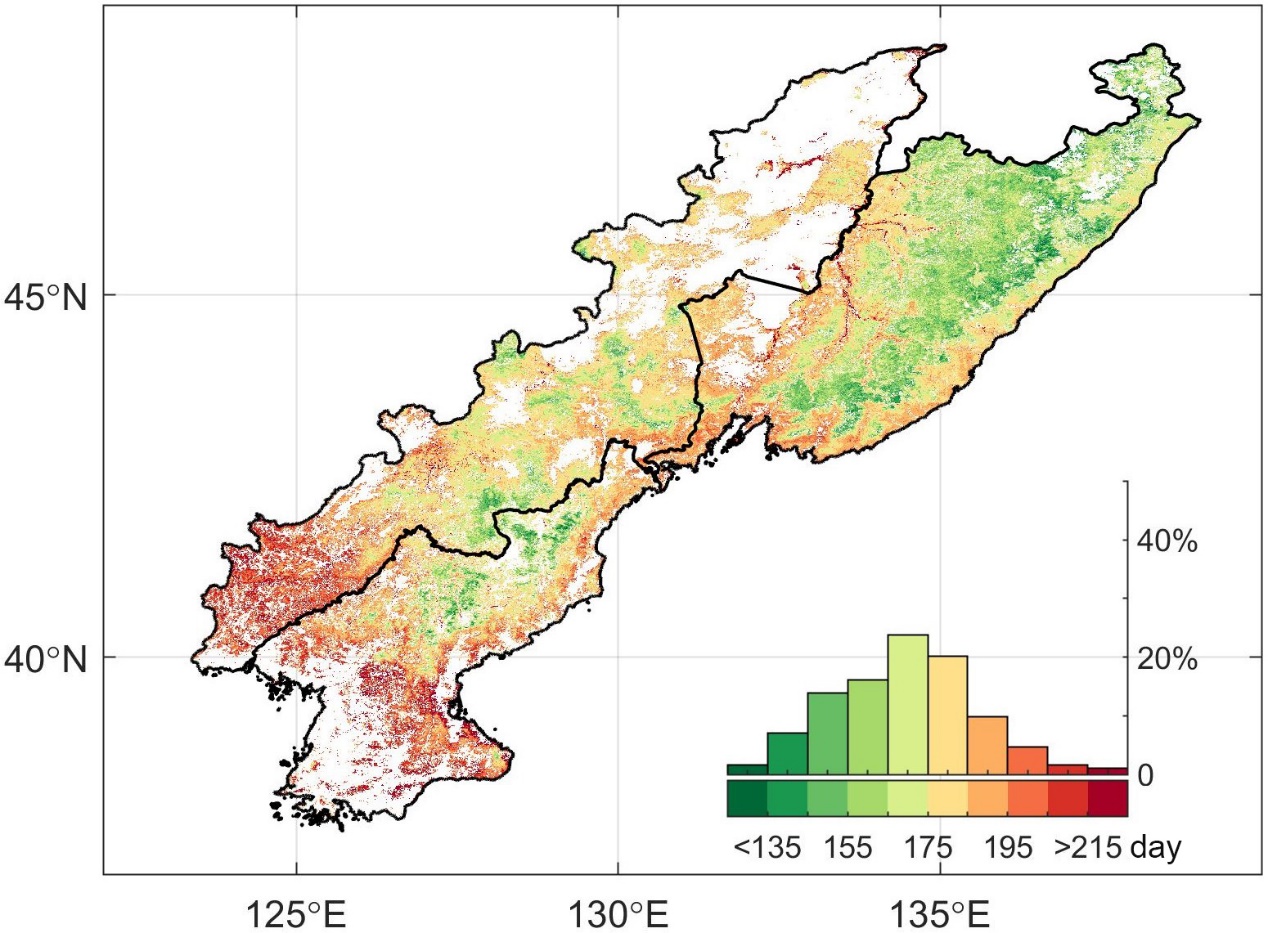
**

**Supplementary Figure 6.** Spatial distribution of length of growing season in the CRCDR from 2001 to 2020.

**
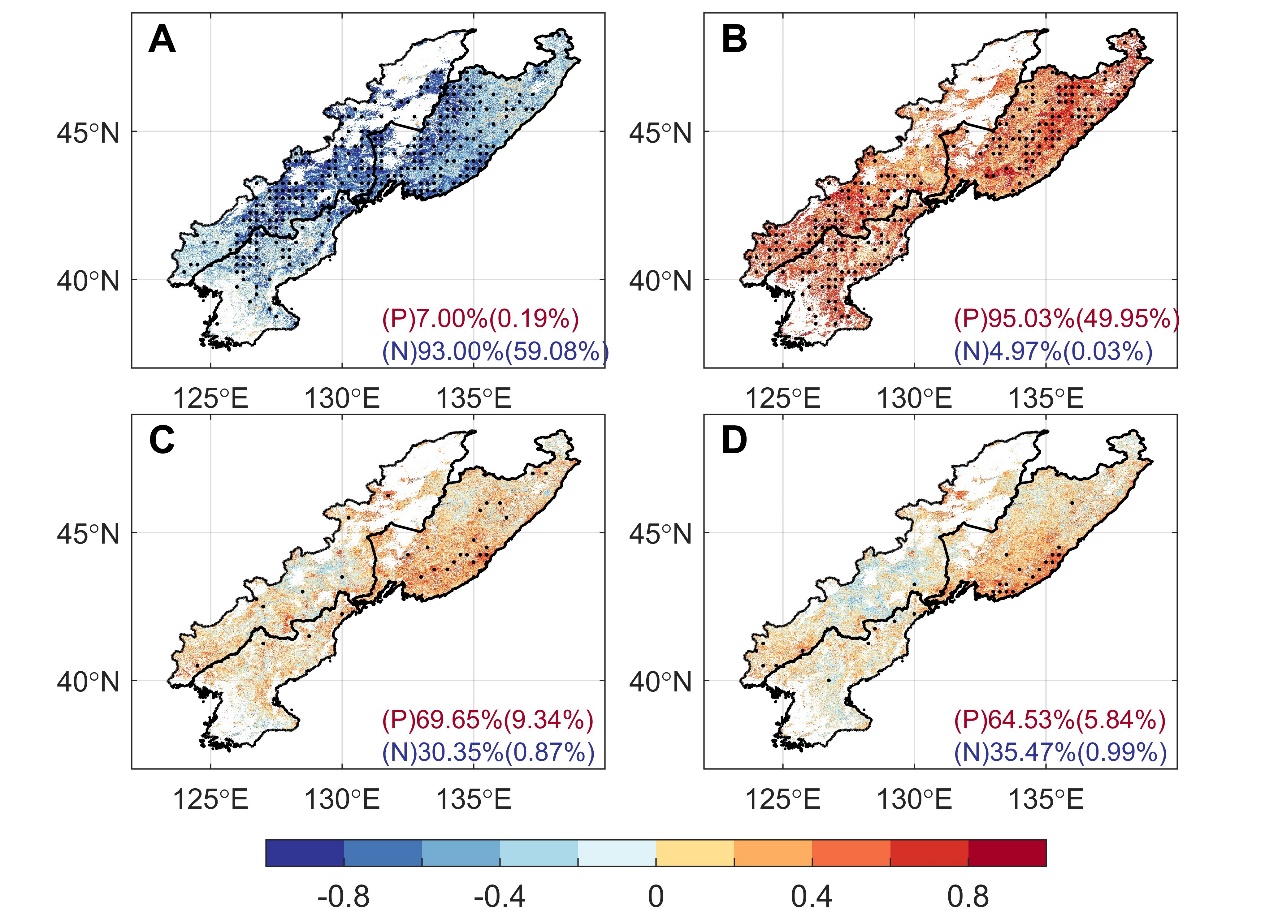
**

**Supplementary Figure 7.** Spatial distribution of correlation coefficients in the first growing season. (A-D) represent SOS, temperature, precipitation, and radiation, respectively. The red "P" and accompanying percentages indicate the proportion of pixels with a positive correlation, with values in parentheses representing the proportion of significant correlations. The blue "N" denotes negative correlations. Black dots represent pixel points with statistically significant correlations (p < 0.05).

**
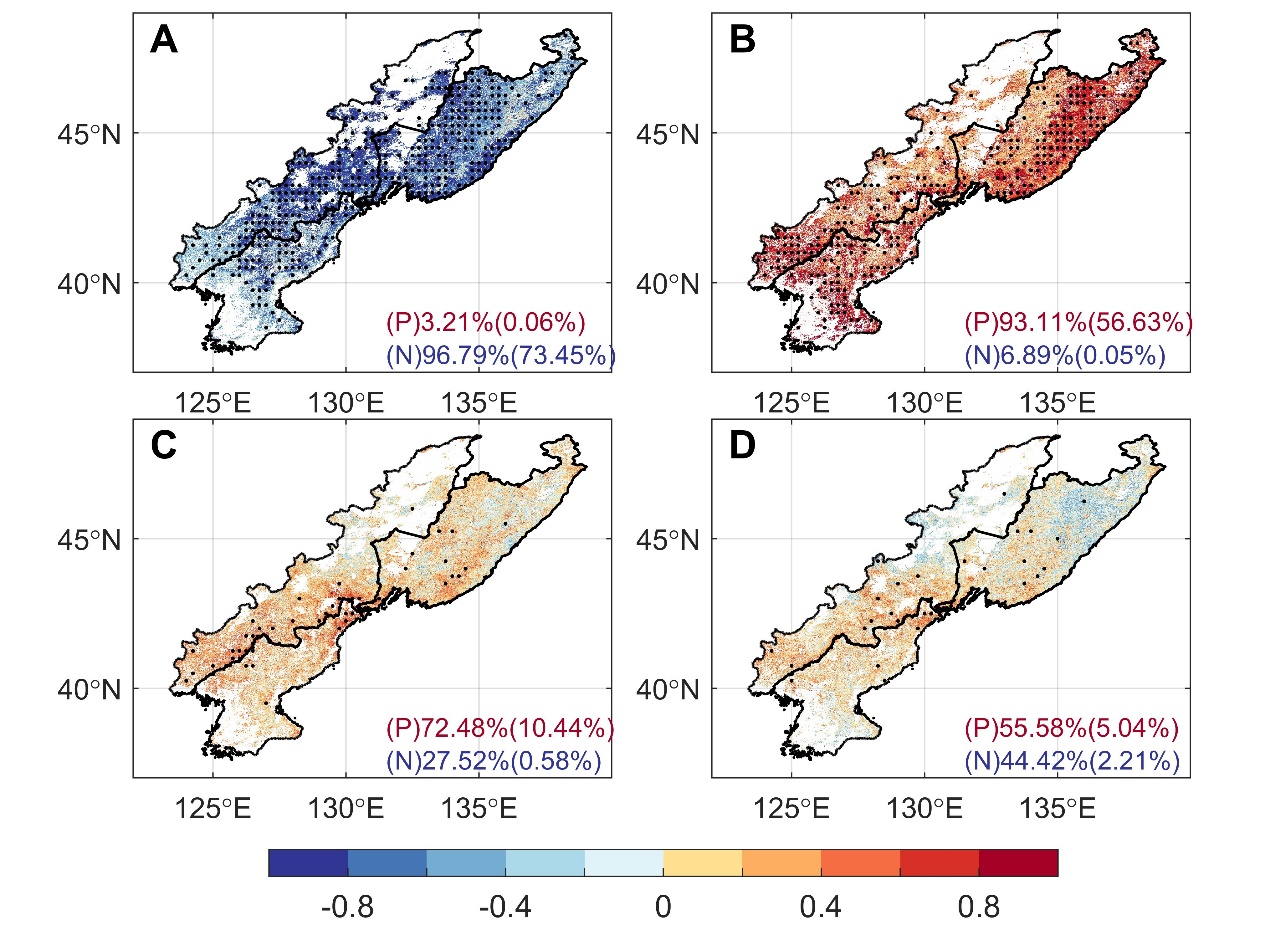
**

**Supplementary Figure 8.** Spatial distribution of correlation coefficients in the second growing season. (A-D) represent SOS, temperature, precipitation, and radiation, respectively. The red "P" and accompanying percentages indicate the proportion of pixels with a positive correlation, with values in parentheses representing the proportion of significant correlations. The blue "N" denotes negative correlations. Black dots represent pixel points with statistically significant correlations (p < 0.05).

**
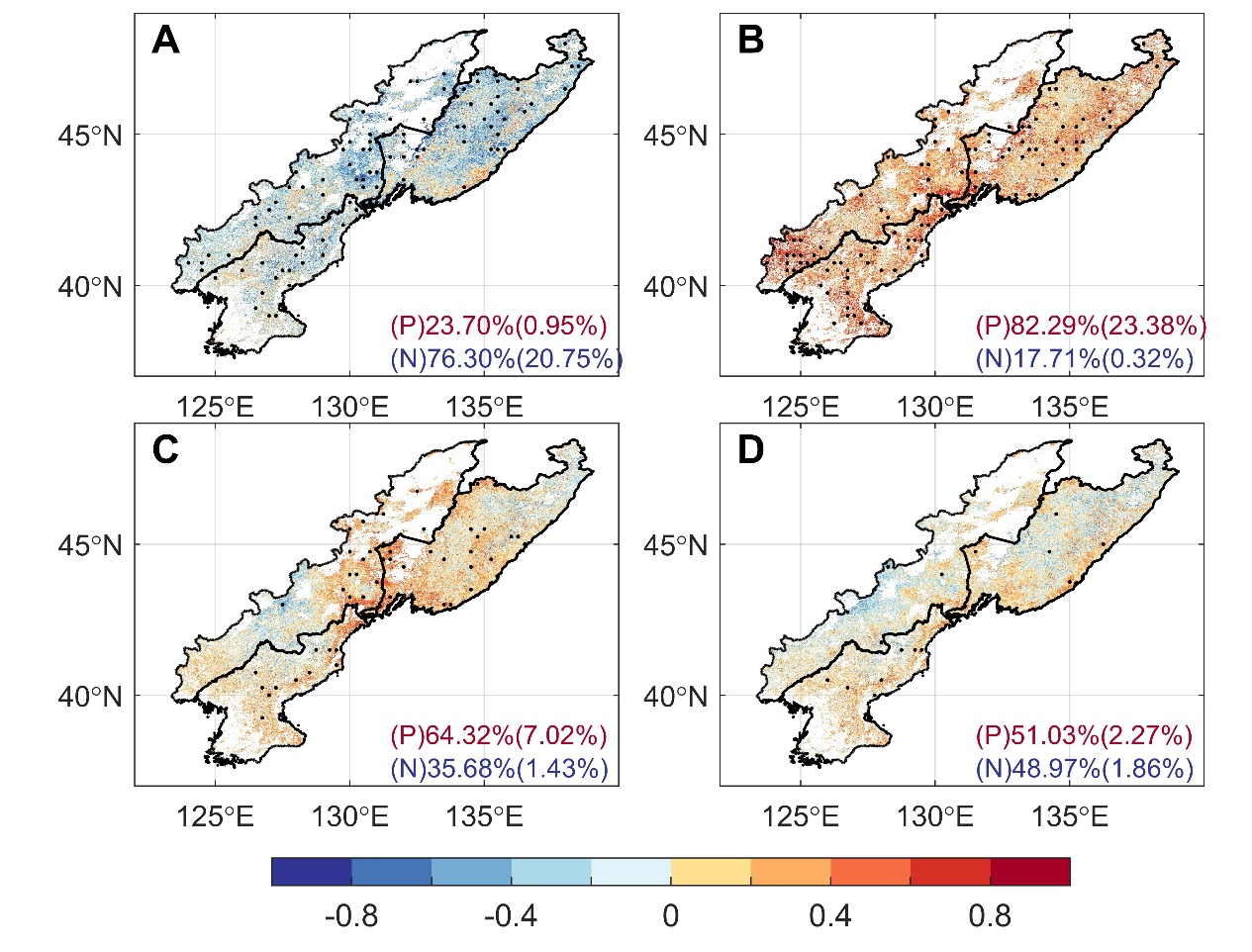
**

**Supplementary Figure 9.** Spatial distribution of correlation coefficients in the third growing season. (A-D) represent SOS, temperature, precipitation, and radiation, respectively. The red "P" and accompanying percentages indicate the proportion of pixels with a positive correlation, with values in parentheses representing the proportion of significant correlations. The blue "N" denotes negative correlations. Black dots represent pixel points with statistically significant correlations (p < 0.05).

**
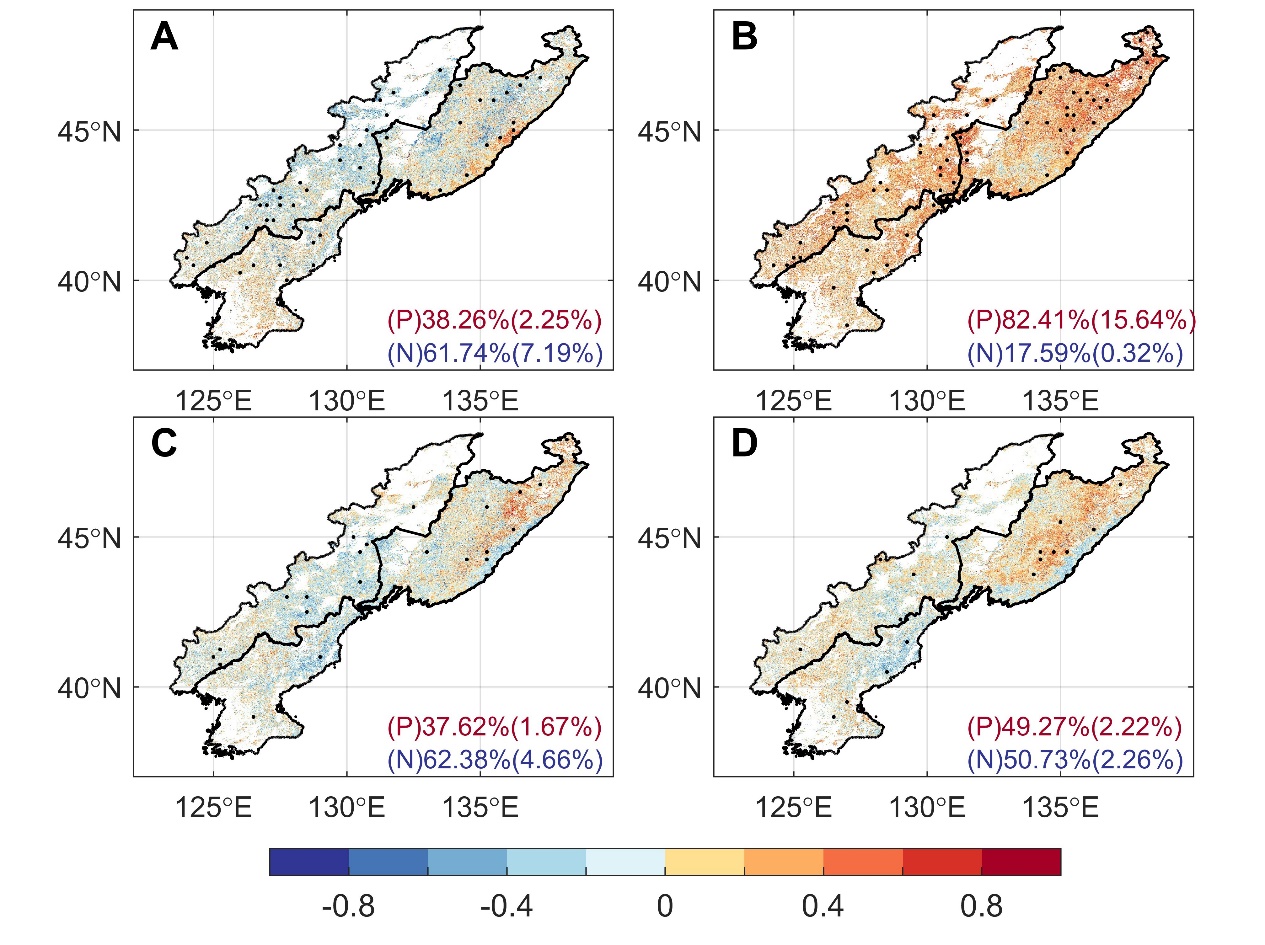
**

**Supplementary Figure 10.** Spatial distribution of correlation coefficients in the fourth growing season. (A-D) represent SOS, temperature, precipitation, and radiation, respectively. The red "P" and accompanying percentages indicate the proportion of pixels with a positive correlation, with values in parentheses representing the proportion of significant correlations. The blue "N" denotes negative correlations. Black dots represent pixel points with statistically significant correlations (p < 0.05).

**
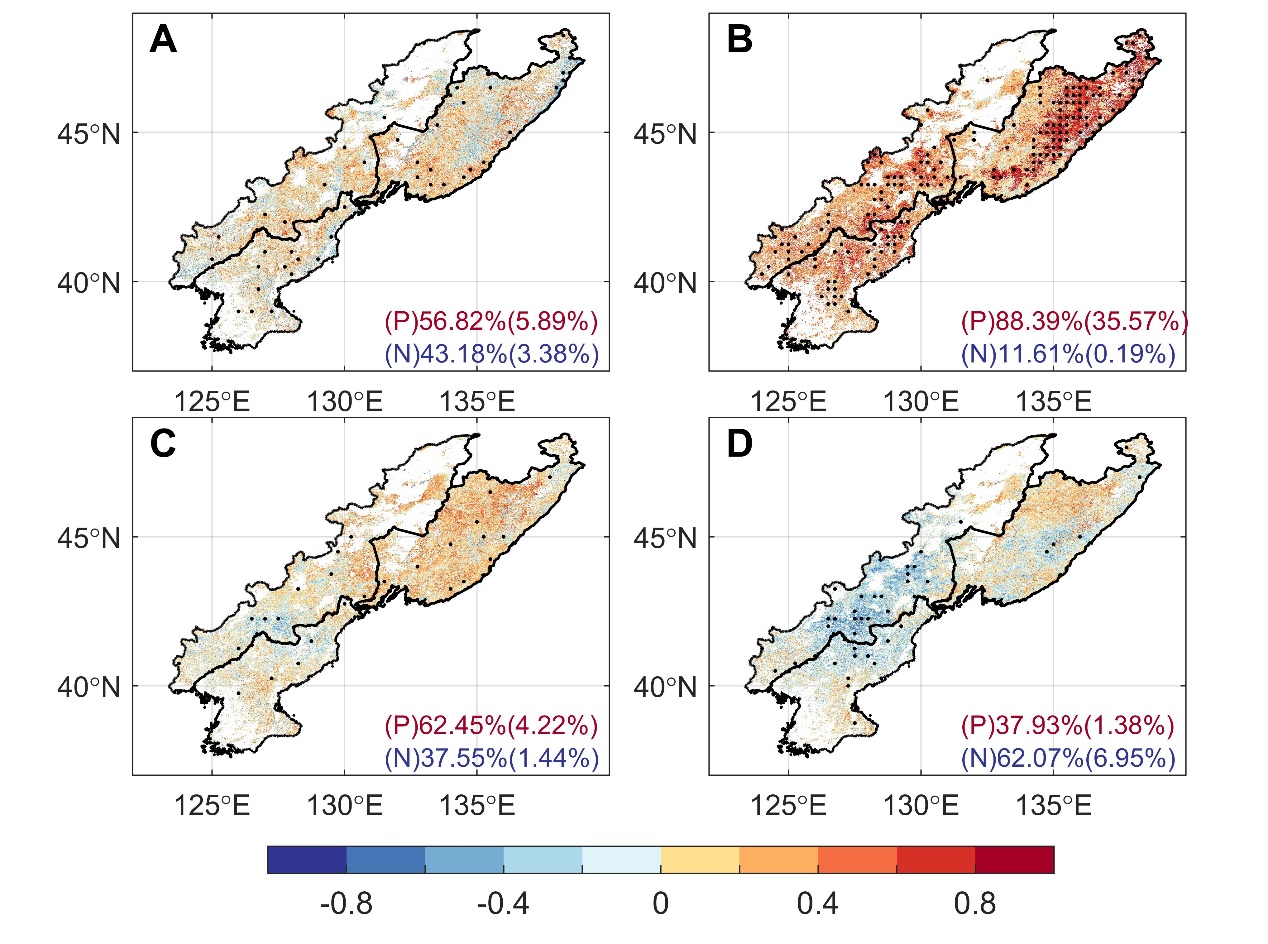
**

**Supplementary Figure 11.** Spatial distribution of correlation coefficients in the fifth growing season. (A-D) represent SOS, temperature, precipitation, and radiation, respectively. The red "P" and accompanying percentages indicate the proportion of pixels with a positive correlation, with values in parentheses representing the proportion of significant correlations. The blue "N" denotes negative correlations. Black dots represent pixel points with statistically significant correlations (p < 0.05).

**
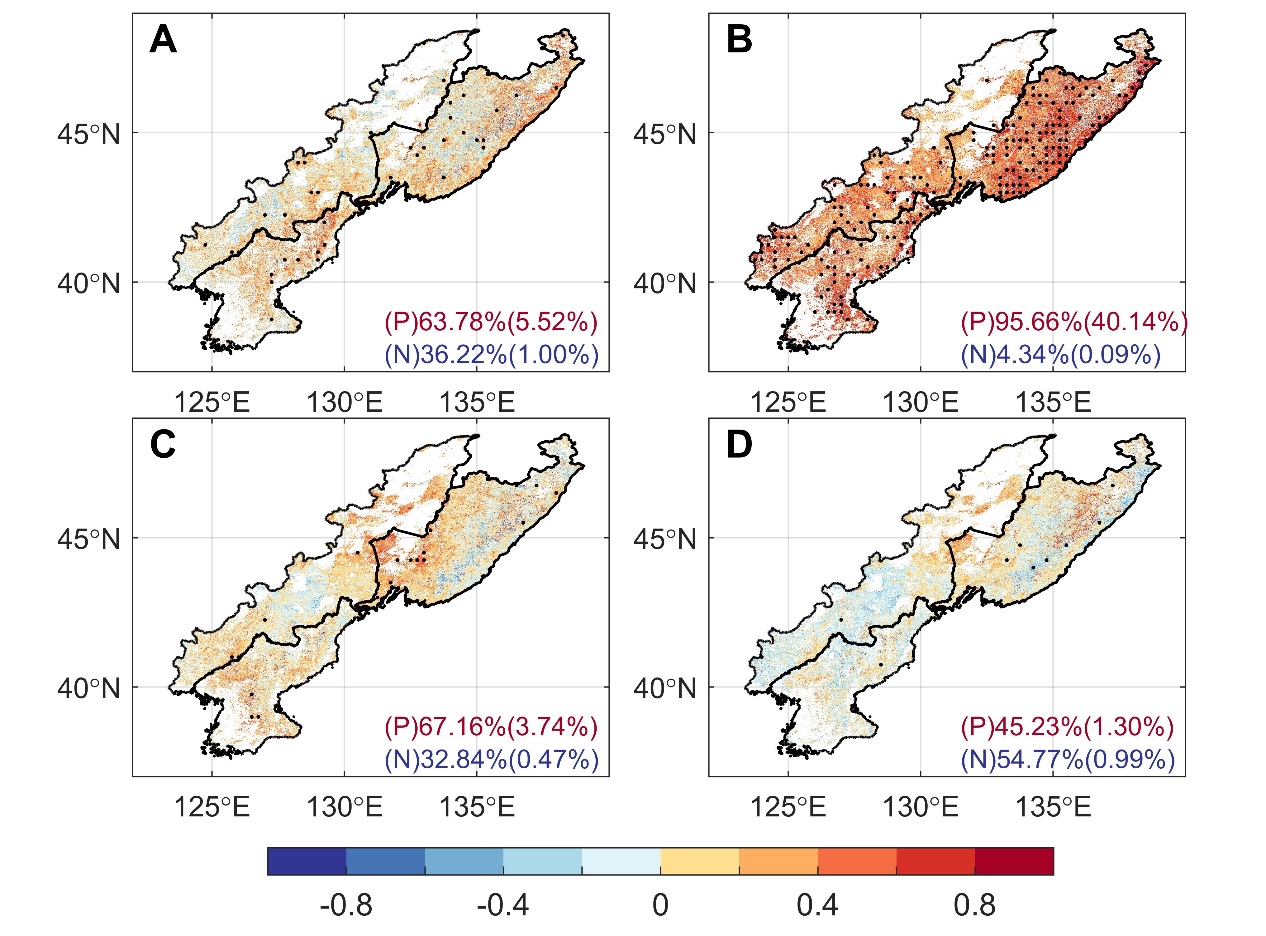
**

**Supplementary Figure 12.** Spatial distribution of correlation coefficients in the sixth growing season. (A-D) represent SOS, temperature, precipitation, and radiation, respectively. The red "P" and accompanying percentages indicate the proportion of pixels with a positive correlation, with values in parentheses representing the proportion of significant correlations. The blue "N" denotes negative correlations. Black dots represent pixel points with statistically significant correlations (p < 0.05).

**

**

**Supplementary Figure 13.** Result of NDVI time series before and after S-G filtering for the main vegetation types in the CRCDR region (the blue curve represents the NDVI time series before filtering, and the red curve represents the NDVI time series after filtering).
